# Prodrug Strategies in Atherosclerosis: Targeted Delivery and Therapeutic Advances

**DOI:** 10.1155/cdr/5159097

**Published:** 2026-08-17

**Authors:** Elaheh Mirhadi, Wael Almahmeed, Prashant Kesharwani, Amirhossein Sahebkar

**Affiliations:** ^1^ Biotechnology Research Center, Pharmaceutical Technology Institute, Mashhad University of Medical Sciences, Mashhad, Iran, mums.ac.ir; ^2^ Heart and Vascular Institute, Cleveland Clinic Abu Dhabi, Abu Dhabi, UAE, clevelandclinicabudhabi.ae; ^3^ Next-Generation Translational Nanomedicine Laboratory, Department of Pharmaceutical Sciences, Dr. Harisingh Gour Vishwavidyalaya (A Central University), Sagar, Madhya Pradesh, India; ^4^ University Institute of Pharma Sciences, Chandigarh University, Mohali, Punjab, India, chandigarhuniversity.ac.in; ^5^ Centre for Research Impact and Outcome, Chitkara College of Pharmacy, Chitkara University, Rajpura, Punjab, India, chitkara.edu.in; ^6^ Applied Biomedical Research Center, Basic Sciences Research Institute, Mashhad University of Medical Sciences, Mashhad, Iran, mums.ac.ir

**Keywords:** atherosclerosis, inflammation, lipid metabolism, oxidative stress, prodrug

## Abstract

Atherosclerosis is an advancing and complex vascular condition primarily associated with sustained inflammatory responses, oxidative imbalance, and disruptions in lipid homeostasis. These pathological features render it a major contributor to the global burden of cardiovascular disease, encompassing both morbidity and mortality. Traditional treatment modalities, including statins, angiotensin‐converting enzyme inhibitors, PCSK9 inhibitors and surgical procedures, frequently encounter limitations due to inefficient pharmacokinetic profiles, insufficient targeting of pathological sites and widespread adverse effects. To address these therapeutic shortcomings, prodrug and nano‐prodrug technologies have gained recognition as innovative systems capable of enabling site‐specific and stimuli‐activated therapeutic delivery in atherosclerotic disease management. This review discusses ROS‐responsive prodrugs, biomimetic nano‐prodrug platforms, and multifunctional theranostic nanotechnologies as alternative approaches for advanced atherosclerosis treatment. These systems enable targeted drug activation in oxidative plaque environments, improve pharmacokinetics, and enhance lesion‐specific delivery via mechanisms such as VCAM‐1 targeting, integrins, and cell membrane cloaking. Notably, in preclinical models nano‐prodrugs have been reported to produce combined therapeutic effects by modulating lipid metabolism, reducing inflammation, and preventing foam cell formation, whereas theranostic platforms have enabled real‐time monitoring of treatment response in animals. No nano‐prodrug platform for atherosclerosis has yet been evaluated in humans. Despite progress, key barriers to clinical translation remain, including poor scalability and reproducibility of nanomaterials, patient variability in plaque biology, limited long‐term safety data, and regulatory challenges for complex nanosystems. Future advances will require biomarker‐guided patient selection, robust clinical validation, multistimuli‐responsive designs, and well‐defined regulatory standards. Overcoming these issues is vital for integrating nano‐prodrug technologies into precision atherosclerosis therapy.

## 1. Introduction

Atherosclerosis, a long‐term condition that impacts both elastic and muscular‐elastic arteries, is pivotal in the development of cardiovascular disease (CVD). The condition is characterized by the development of fibrofatty lesions within the walls of arteries, leading to considerable morbidity and mortality on a global scale, encompassing the majority of myocardial infarctions and numerous strokes [1]. Atherosclerosis develops due to multiple factors that lead to the formation of fibrous plaques, such as diets high in lipoproteins, smoking, diabetes, hypertension, and viral infections, all of which amplify chronic arterial conditions [2, 3]. Early identification of atherosclerosis is critical for preventing CVD progression and reducing global health burden. Atherosclerosis begins in childhood and develops over decades before clinical manifestations appear in middle age [4]. Timely diagnosis in clinical cardiology is essential to prevent complications and maintain patients′ health and functionality [5]. Advancements in noninvasive diagnostic methods, including high‐resolution imaging and new biomarkers, now allow for detecting early‐phase lesions, even in asymptomatic individuals [6].

**Figure 1:**
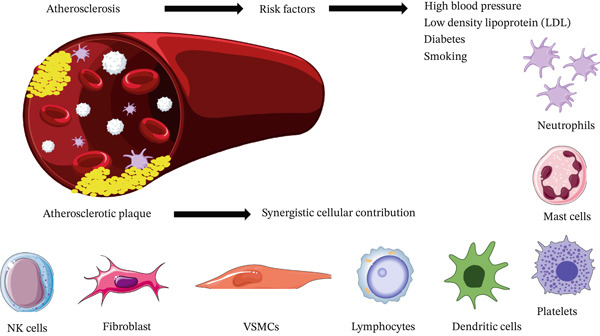
Overview of atherosclerosis pathogenesis, highlighting key cellular contributors and major risk factors involved in plaque formation and arterial wall remodeling.

Atherosclerosis develops within a complex and constantly evolving vascular microenvironment where various cell types play distinct interconnected roles [7]. Endothelial cells (ECs), which line the vessel wall, become dysfunctional under oxidative stress, increasing permeability to lipids and expressing adhesion molecules that recruit circulating monocytes [8]. These monocytes differentiate into macrophages within the intima, where they internalize modified lipids, transform into foam cells [9, 10]. Vascular smooth muscle cells (VSMCs) contribute by migrating from the media to the intima, proliferation, and production of extracellular matrix components, while also having the capacity to adopt macrophage‐like phenotypes and participate in lipid uptake [11–13]. Meanwhile, activated lymphocytes, in particular T cells, modulate immune responses through secreting cytokines that in consequence regulate macrophage activity and vascular inflammation [8, 14–16]. Altogether, these cells form a highly coordinated network driven by lipid accumulation, oxidative stress, and signaling cascades, ultimately resulting in plaque growth and instability. The spatial and temporal interplay among these processes adds substantial complexity, posing a major challenge for the development of effective and targeted therapies (Figure 1).

Conventional drug therapies, although clinically useful, do not fully address the complexity of atherosclerosis. Many compounds have poor penetration to the arterial intima, limiting their interaction with cells within the plaque [17, 18]. Their relatively short circulation time and rapid elimination further decrease effective concentrations at diseased sites, whereas nonspecific distribution could contribute to the systemic adverse effects and reduced therapeutic efficiency. Moreover, these treatments are not optimized for the localized oxidative and inflammatory milieu of atherosclerotic lesions, and therefore have limited impact on critical processes such as [19, 20].

These challenges highlight the need for more advanced therapeutic approaches that can precisely act within the atherosclerotic microenvironment. Prodrug and nano‐prodrug approaches address limitations through enabling stimuli‐responsive activation such as reactive oxygen species (ROS), enzymatic activity, acidic conditions and via directing drugs to key cellular players in plaque formation, including ECs, macrophages, and inflamed vascular sites. Incorporating biomimetic surfaces, targeting ligands, and controlled release features, these systems are manufactured to bypass biological barriers, enhance penetration into the vessel wall, and reduce off‐target toxicity [21, 22].

This review brings together prodrug and nano‐prodrug approaches addressing the cellular and inflammatory complexity of atherosclerosis, complementing recent reviews of nanoparticle delivery, stimulus‐responsive nanomedicine and ROS‐based nanotherapeutics by focusing specifically on prodrug activation chemistry. It focuses on recent advancements in ROS‐responsive systems, biomimetic nanocarriers, and multifunctional theranostic systems, highlighting their ability for precise, site‐specific drug activation and their promise for improved clinical application.

### 1.1. Role of Inflammation in Atherosclerosis

**Figure 2:**
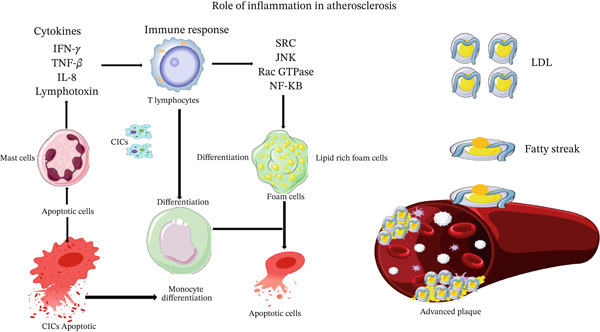
Illustration of immunoinflammatory pathways involved in the progression of atherosclerosis. The schematic highlights the sequential recruitment of monocytes, their differentiation into macrophages, and subsequent transformation into foam cells through the internalization of oxidized low‐density lipoprotein (ox‐LDL). Principal immune cell populations such as T lymphocytes, mast cells, and macrophages mediate the release of proinflammatory cytokines (e.g., IFN‐*γ*, TNF‐*β*, IL‐8) and facilitate extracellular matrix remodeling via MMPs. CD36‐associated signaling pathways (including SRC, JNK, Rac GTPase, and NF‐*κ*B) are implicated in the induction of oxidative stress and dysregulated lipid metabolism, ultimately contributing to the emergence of fatty streaks, structural plaque instability, and thrombotic events.

Inflammation plays a fundamental role in the initiation and progression of atherosclerosis, alongside lipid accumulation, marking a shift from purely lipid‐centered concepts of the disease [23]. Atherogenic risk factors trigger immune‐mediated responses that promote vascular dysfunction and chronic inflammation [24]. Monocytes, T lymphocytes, and mast cells are recruited to the intimal layer of the vascular wall through adhesion molecule‐mediated mechanisms. Upon activation by inflammatory stimuli, T cells secrete key cytokines, including interferon‐*γ* (IFN‐*γ*), lymphotoxin, and tumor necrosis factor‐*β* (TNF‐*β*), thereby amplifying the local immune response (Figure 2) [25, 26].

As the disease progresses, persistent inflammation leads to increased oxidative stress and further modification of lipoproteins, enhancing leukocyte recruitment and sustaining a proinflammatory environment. In advanced plaques, macrophages and T cells secrete matrix‐degrading enzymes, including matrix metalloproteinases (MMPs), which weaken the fibrous cap. This structural destabilization increases the risk of plaque rupture, thrombosis, and subsequent arterial obstruction [27–30].

CD36 and its associated ligands, including modified low‐density lipoprotein (mLDL), high‐density lipoprotein (HDL), fatty acids, and very‐low‐density lipoprotein (VLDL), play a vital role in lipoprotein internalization and the regulation of lipid metabolism within the vascular wall [31]. The interaction between CD36 and modified lipoproteins promotes the uptake of oxidized LDL by macrophages, leading to lipid accumulation and foam cell formation, a defining feature of early atherogenesis. This process contributes directly to the expansion of fatty streaks and the progression of atherosclerotic lesions through sustained intracellular lipid deposition [32, 33].

Beyond its role in lipid handling, CD36 also functions as a key mediator of proinflammatory signaling. Binding of mLDL to CD36 activates multiple downstream signaling pathways, including c‐Jun N‐terminal kinase (JNK), Rac GTPase, nonreceptor tyrosine kinase (SRC), and the nuclear factor‐kappa B (NF‐*κ*B) transcription factor. Activation of these pathways promotes oxidative stress and induces the proinflammatory cytokines expression, thereby amplifying the inflammatory microenvironment within atherosclerotic plaques and in consequence accelerating disease progression [34, 35].

Collectively, inflammatory signaling pathways implicated in atherosclerosis not only contribute to the progression of the disease but also provide well‐defined targets for therapeutic intervention through prodrug‐based approaches [8]. Enzyme‐responsive strategies are particularly significant, as proteolytic enzymes including MMPs, cathepsins, and legumain are overexpressed in inflamed atherosclerotic plaques and can be leveraged to facilitate site‐specific activation of prodrugs [36]. Concurrently, radical‐responsive systems, especially those employing ROS‐sensitive linkers, allow for controlled drug release within the oxidative milieu characteristic of atherosclerotic lesions, thereby improving targeting accuracy while reducing off‐target systemic effects [37].

Receptor‐mediated targeting represents an effective approach for directing prodrugs towards the specific cellular populations that drive plaque formation. Scavenger receptors such as CD36, adhesion molecules like vascular cell adhesion molecule‐1 (VCAM‐1), and integrin receptors present on activated ECs and macrophages can be exploited to promote selective cellular uptake and subsequent intracellular activation of prodrug platforms [38, 39]. Together, these targeting strategies establish a mechanistic connection between the immunoinflammatory features of atherosclerosis and the rational development of advanced prodrug and nano‐prodrug therapeutics.

### 1.2. Role of Oxidative Stress in Atherosclerosis

Oxidative stress is pivotal in the development and progression of atherosclerosis, primarily due to the excessive production of ROS that surpasses the capacity of antioxidant defense mechanisms [40]. Oxidative stress and chronic inflammation play central roles in atherogenesis by promoting cellular proliferation, hypertrophy, apoptosis, and activation of Rho kinase through intracellular signaling pathways or direct endothelial damage [41]. These processes are tightly interconnected, each potentiating the other in a self‐reinforcing cycle. Increasing evidence indicates that endothelial dysfunction, in concert with oxidative and inflammatory stress, establishes a positive feedback loop that drives and sustains atherosclerotic progression, ultimately contributing to unfavorable cardiovascular outcomes [42]. Notably, the elevated concentration of ROS within atherosclerotic plaques creates a distinctive microenvironment that can be leveraged for targeted therapeutic strategies. Oxidized LDL (oxLDL) promotes apoptosis in various cell types; however, it has been noted that a significant quantity of oxidized LDL builds up in macrophages, resulting in the formation of foam cells without triggering apoptosis [43, 44]. oxLDL contributes significantly to the early development, sustained presence, and advancement of atherosclerotic lesions. The lectin‐like oxidized LDL receptor‐1 (LOX‐1), identified as a newly characterized scavenger receptor for oxLDL, was originally isolated from vascular ECs [45]. New insights have been revealed regarding the mechanisms that contribute to the formation and progression of carotid atherosclerosis, which may help identify potential therapeutic targets for treatment [46].

Enhanced expression of ROS scavenging systems has been shown to attenuate or stabilize atherosclerotic plaque formation, with outcomes influenced by the genetic profile of the murine model. In human subjects, elevated concentrations of circulating oxidative biomarkers originating from protein oxidation (e.g., oxidized LDL, nitrotyrosine, protein carbonyls, and advanced glycation end‐products) and lipid peroxidation (e.g., 8‐epi‐prostaglandin F2*α* and malondialdehyde) are commonly observed in individuals with elevated cardiovascular risk or clinically manifest atherosclerotic cardiovascular disease (ASCVD). Notably, several of these biomarkers have emerged as independent prognostic indicators of ASCVD in extensive population‐based studies [47].

This oxidative setting serves as an intrinsic biochemical stimulus for site‐specific prodrug activation, allowing selective drug release at diseased vascular regions. In particular, ROS‐responsive linkers including disulfide, thioketal, and boronate bonds are susceptible to cleavage under high ROS conditions, enabling controlled drug release within inflamed, oxidative tissues [48, 49]. This strategy provides a rational basis for designing advanced stimuli‐responsive prodrug systems with improved targeting accuracy and minimized systemic toxicity [50].

Oxidative stress and inflammation in atherosclerosis are not discrete entities; rather, they represent tightly coupled and mutually reinforcing processes that drive and sustain chronic vascular injury. Elevated levels of ROS promote the activation of inflammatory pathways, whereas infiltrating immune cells further amplify oxidative stress, thereby facilitating disease progression. This reciprocal and highly integrated pathological relationship underscores a strong rationale for the development of stimuli‐responsive prodrug systems capable of exploiting both oxidative and inflammatory cues to enable targeted therapeutic delivery.

## 2. Conventional Treatments

Current management strategies for atherosclerosis encompass both interventional techniques and pharmacological treatments [51, 52]. Surgical methods, including coronary artery bypass grafting, angioplasty, and percutaneous coronary intervention, are successful in reestablishing vascular patency; nevertheless, they are invasive and carry risks such as hemorrhage and the formation of subcutaneous hematomas [53–55].

Pharmacological management primarily focuses on reducing systemic lipids, with statins serving as the foundational treatment due to their significant ability to lower LDL‐C and their additional anti‐inflammatory and antioxidant properties [56–60]. For patients who fail to reach the desired LDL‐C levels or who cannot tolerate statins, supplementary treatments such as ezetimibe or PCSK9 inhibitors may be utilized [61–63]. Other nonstatin medications, such as fibrates [64], niacin, and CETP inhibitors, have demonstrated inconsistent or limited clinical advantages and are not generally recommended because of their variable efficacy and unfavorable risk‐benefit ratios [65, 66].

Despite these advancements, current pharmacotherapies demonstrate several inherent limitations that hinder their overall efficacy in managing atherosclerosis. Firstly, the majority of agents lack precise targeting abilities and operate systemically instead of selectively accumulating within atherosclerotic plaques [19, 67]. This nonspecific distribution diminishes local drug concentrations at the pathological site while heightening the risk of off‐target effects and systemic toxicity. Secondly, numerous conventional medications possess relatively short half‐lives, which necessitate frequent administration and may compromise patient adherence. Lastly, the intricate structure of advanced atherosclerotic lesions, especially fibrous plaques, poses a considerable obstacle to drug penetration, thereby limiting therapeutic effectiveness at crucial sites [37, 68].

Moreover, although anti‐inflammatory approaches have attracted considerable interest, currently available agents including cytokine‐targeting therapies and pathway‐specific inhibitors have yielded modest efficacy and, in certain cases, unfavorable cardiovascular outcomes, as reported with TNF‐*α* and COX‐2 inhibitors [69, 70]. Taken together, these limitations indicate that existing treatments, despite their ability to regulate systemic risk factors, fall short in providing targeted, durable, and plaque‐specific therapeutic effects. This highlights the necessity for next‐generation strategies designed to overcome biological barriers, enhance lesion‐specific delivery, and reduce systemic toxicity.

Prodrug‐based approaches provide a logical solution by structurally modulating active therapeutic agents into biologically inert or less active precursors, which could be activated within the pathological microenvironment. This concept has been extensively studied in the context of chemotherapeutic agents in cancer [71–73], and has been extended to other pathological states such as atherosclerosis [74, 75]. This strategy enables improved pharmacokinetic functions, such as enhanced stability and extended circulation time, while facilitating targeted activation in response to disease‐specific stimuli such as ROS, pH changes, and enzymatic activity as well. Notably, prodrug systems significantly decrease systemic drug exposure, which consequently minimizes off‐target toxicity and improves therapeutic safety [76, 77]. By using nanocarrier‐based delivery platforms, these privileges are further reinforced through enhanced vascular wall penetration, augmented plaque accumulation, and controlled drug release. Collectively, prodrug approaches directly eliminate the major drawbacks of conventional therapies including limited bioavailability, poor targeting, and systemic side effects offering a more accurate and efficient strategy for the treatment of atherosclerosis [78].

## 3. Prodrug Strategies in Atherosclerosis

**Figure 3:**
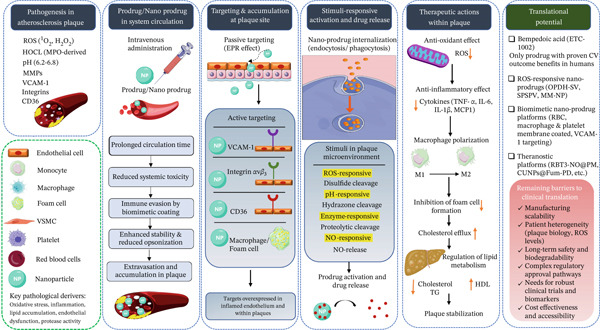
Schematic overview of targeted prodrug and nano‐prodrug strategies in atherosclerosis. The illustration summarizes plaque pathogenesis, systemic circulation of nano‐prodrugs, endothelial and macrophage‐mediated targeting, stimuli‐responsive activation (ROS, pH, enzymes, NO), therapeutic actions including lipid regulation, inflammation reduction, and foam‐cell inhibition, and key translational considerations for clinical development.

Prodrug‐based strategies have emerged as a promising approach to enhance the therapeutic efficacy and specificity of agents used in atherosclerosis (Figure 3). Prodrugs are pharmacologically inactive forms of active compounds that are designed to overcome limitations of the parent drug, including toxicity, poor stability, low solubility, and lack of targeted delivery. Most cardiovascular drugs are subject to extensive first‐pass metabolism, resulting in reduced activity and the formation of potentially harmful metabolites, making them suitable candidates for prodrug‐based modification. Because oxidative stress, inflammation, and endothelial dysfunction are key drivers of atherogenesis, modern prodrug strategies increasingly aim for site‐specific activation within affected vascular tissues [79].

### 3.1. Conventional Prodrug Approaches

Traditional prodrug strategies in atherosclerosis have primarily focused on improving stability, bioavailability, and safety profiles of existing therapeutics [80].

A notable example is aspirin eugenol ester (AEE), generated through esterification of aspirin and eugenol. Through strategic esterification, the carboxylic acid moiety of aspirin and the phenolic hydroxyl group of eugenol were chemically masked to generate AEE, following the prodrug design approach. This modification enhanced the compound′s structural stability and mitigated gastrointestinal side effects. After administration, AEE undergoes metabolic conversion into its parent drugs, enabling a synergistic therapeutic effect against atherosclerosis. In a hamster model of diet‐induced atherosclerosis, AEE treatment led to a marked reduction in body weight gain, normalization of lipid parameters (triglycerides, total cholesterol, and LDL), and attenuation of hepatic steatosis and vascular lesions. Metabonomic profiling using UPLC‐Q‐TOF/MS revealed distinct plasma and urinary biomarkers linked to lipid, amino acid, and energy metabolism, underscoring AEE′s modulatory role in correcting metabolic imbalances associated with atherosclerotic progression [81].

Bempedoic acid (ETC‐1002) represents another clinically relevant prodrug targeting ATP citrate lyase (ACLY). ETC‐1002 functions as a hepatoselective prodrug targeting ACLY, an upstream regulator of HMG‐CoA reductase within the cholesterol biosynthesis cascade [82]. Following activation in hepatocytes via very long‐chain acyl‐CoA synthetase‐1 (ACSVL1), its active metabolite ETC‐1002‐CoA limits the generation of acetyl‐CoA, thereby curbing the synthesis of cholesterol and fatty acids [83]. In contrast to statins, its liver‐specific activation prevents skeletal muscle exposure, thus mitigating myotoxic effects [84]. In mice deficient for both ApoE and AMPK*β*1, administration of ETC‐1002 led to a marked reduction in hepatic cholesterol and triglyceride levels, downregulated lipogenic activity, and mitigated the progression of atherosclerotic lesions through mechanisms independent of AMPK signaling [85]. Preclinical investigations revealed robust lipid‐lowering, anti‐inflammatory, and antiatherosclerotic properties, as evidenced by enhanced M2 macrophage polarization and reduced aortic plaque burden [86]. ETC‐1002 is a hepatoselective prodrug targeting ACLY and is the only prodrug in this review with demonstrated cardiovascular benefit in humans. In the CLEAR Outcomes trial [87], 13,970 statin‐intolerant patients were randomized and bempedoic acid significantly reduced major adverse cardiovascular events, leading to FDA approval for cardiovascular risk reduction [87].

In individuals suffering from chronic inflammatory disorders and those who have undergone transplantation populations often managed with immunosuppressive medications like azathioprine [88]. Azathioprine acts as a prodrug that is swiftly transformed into 6‐mercaptopurine (6‐MP), a substance recognized for its immunomodulatory and antiproliferative effects. Nevertheless, the precise influence of this compound on the advancement of atherosclerosis remains inadequately clarified [89]. The antiatherogenic properties of 6‐MP have been explored with particular emphasis on its modulatory impact on monocytes and macrophages critical cellular contributors to plaque formation. Findings indicated that 6‐MP promotes apoptosis in THP‐1 monocytes by suppressing the expression of key intrinsic antiapoptotic regulators, namely Bcl‐2 and Bcl‐XL. In addition, it impairs monocyte adhesion through downregulation of adhesion molecules such as PECAM‐1 and VLA‐4. Notably, 6‐MP also markedly inhibited both the transcription and secretion of monocyte chemoattractant protein‐1 (MCP‐1) in activated macrophages, a chemokine essential for monocyte recruitment during atherogenesis [90].

Secretory phospholipase A2 (sPLA2) enzymes are critically involved in regulating lipid metabolism and mediating inflammatory responses, both of which are central to the development of atherosclerosis and vascular aneurysms [91]. Increased expression of sPLA2 isoforms IIA, V, and X has been documented in aortic lesions of both human and murine origin, where these enzymes facilitate lipoprotein alteration, foam cell generation, and inflammatory activation within the vascular wall [92]. Accordingly, pharmacological inhibition of sPLA2 activity was explored as a therapeutic strategy in CVD. However, this approach failed in clinical testing. In the phase III VISTA‐16 trial [93], 5145 patients with acute coronary syndrome were randomized to varespladib or placebo on top of atorvastatin. The study was terminated early for futility and possible harm, with a significant excess of myocardial infarction in the varespladib group (3.4% vs 2.2%; HR 1.66, 95% CI 1.16–2.39). These findings led the authors to conclude that sPLA2 inhibition may be harmful and is not a useful strategy, and the field was subsequently abandoned. The preclinical data below should therefore be interpreted in light of this outcome. Varespladib methyl (A‐002), the orally administered prodrug of A‐001, was originally developed through a structure‐guided design to achieve potent suppression of sPLA2 enzymatic activity, and showed favorable anti‐inflammatory and antiatherosclerotic effects in animal models. Its clinical failure highlights the translational gap between mechanistic promise and patient outcomes [94].

CDX‐085 is a water‐soluble prodrug of astaxanthin designed to overcome the poor oral bioavailability of its parent compound. Following gastrointestinal hydrolysis, CDX‐085 liberates active astaxanthin, which is then integrated into circulating lipoproteins (LDL, HDL, VLDL) and disseminated to vascular tissues. This prodrug strategy enables targeted antioxidant enrichment of lipoproteins and vascular membranes, offering a promising approach to mitigate oxidative stress and lipid‐driven inflammation in atherosclerosis [95].

Simvastatin (SV) and lovastatin are themselves lactone prodrugs, hydrolyzed in vivo to their active hydroxy‐acid forms. Separately, and independently of their prodrug nature, they have been reported to modulate the assembly of cholesterol‐like particles in vitro by a nonenzymatic mechanism; whether this contributes to atheroprotection in vivo is unknown. Through application of a plaque array assay integrating flow cytometry with fluorescent‐tagged cholesterol aggregates, the study revealed that these compounds can directly alter the assembly of low‐density (LD‐CP) and high‐density cholesterol‐like particles (HD‐CP) in both buffer systems and human serum, irrespective of HMG‐CoA reductase inhibition. Notably, SV and lovastatin promoted HD‐CP generation while concurrently diminishing LD‐CP formation, indicating a physicochemical interaction with cholesterol substrates [96].

Menini et al. investigated D‐carnosine octylester, an orally bioavailable prodrug designed to overcome the rapid degradation of L‐carnosine by carnosinase. Upon oral administration, D‐carnosine octylester was effectively hydrolyzed by esterases, resulting in the release of active D‐carnosine, which serves as a powerful scavenger of reactive carbonyl species (RCS) such as 4‐hydroxy‐2‐nonenal (HNE). This prodrug approach facilitated the systemic administration of a carnosinase‐resistant RCS quencher, providing a focused method to disrupt carbonyl‐induced inflammatory pathways in both vascular and renal tissues [97].

### 3.2. Stimuli‐Responsive Prodrug Systems

To address the limitations of conventional approaches, stimuli‐responsive prodrugs have been developed to enable site‐specific activation in response to pathological features including oxidative stress, enzymatic activity, or pH changes [98].

Prodrug and nano‐prodrug approaches for atherosclerosis present several significant benefits that collectively mitigate the drawbacks of traditional therapies. These advantages encompass improved accumulation at lesion sites, enhanced pharmacokinetic stability, decreased systemic toxicity, and the capacity for stimulus‐responsive drug activation within the diseased vascular microenvironment. Moreover, various advanced platforms integrate biomimetic or ligand‐mediated designs that facilitate immune evasion and enable targeted delivery to inflamed macrophages, ECs, and lipid‐laden plaques. These overarching principles form the foundation for the numerous platforms examined in the subsequent subsections, which are categorized based on activation mechanisms, targeting strategies, and delivery modalities.

The advancement of a prodrug approach shows potential for improving the pharmacokinetic effectiveness of medications, especially in targeted drug delivery [99]. ROS are critically implicated in the development of atherosclerotic lesions by mediating oxidative stress, inflammation, and apoptosis, which collectively contribute to plaque initiation and progression [100, 101]. ROS‐responsive prodrugs exhibit substantial therapeutic promise for the selective treatment of atherosclerosis, as they enable increased drug release triggered by elevated reactive oxygen species levels within pathological sites [102, 103]. Qu et al. developed a macrophage‐membrane‐functionalized, ROS‐activatable nanoprodrug (MM‐NP) with dual therapeutic capability. The nanoplatform was strategically designed based on a prodrug architecture, in which the therapeutic agent remains pharmacologically inactive during systemic circulation. Activation is specifically triggered by heightened concentrations of ROS within the oxidative milieu of inflamed atherosclerotic plaques. A ROS‐responsive linker facilitated localized therapeutic activation, thereby reducing systemic side effects. To enhance targeting efficiency, the nanoparticle core was cloaked with a macrophage‐derived membrane, which promotes immune system bypass and directed accumulation at vascular lesions through interaction with endothelial adhesion markers. Following activation, the nanoprodrug liberates therapeutic compounds that concurrently inhibit proinflammatory mediators and promote lipid homeostasis. This dual effect was mediated, in part, by the upregulation of cholesterol‐exporting proteins, notably ATP‐binding cassette transporters ABCA1 and ABCG1 [104].

A nano‐prodrug responsive to ROS, termed OPDH‐SV, was engineered through the covalent attachment of SV to a zwitterionic polymer using oxalate‐based linkers. The resulting formulation exhibited notable physicochemical stability and minimal cytotoxicity, while effectively attenuating intracellular ROS levels and lipid accumulation, key factors in suppressing foam cell development. Furthermore, OPDH‐SV facilitated transcellular migration via an endocytosis‐efflux pathway. In vivo assessments validated its extended systemic circulation, site‐specific accumulation, and therapeutic potential in mitigating atherosclerotic progression [105]. In response to the therapeutic demand for early‐phase, lesion‐targeted treatment, a ROS‐activated prodrug micelle (SPCPV) was developed to facilitate selective transport and oxidative stress‐triggered drug release at sites of vascular inflammation. The micellar system was synthesized through covalent attachment of SV to a polymeric scaffold via ROS‐cleavable peroxalate linkers, resulting in a prodrug that remains therapeutically inactive under physiological conditions. Upon exposure to elevated ROS within the oxidative milieu of atherosclerotic lesions, the micelle underwent swift degradation, thereby liberating bioactive SV. Additionally, SPCPV was engineered to selectively recognize VCAM‐1, which is upregulated in inflamed ECs, facilitating targeted deposition at diseased vascular sites [74]. Moreover, SV was covalently linked to *α*‐tocopherol–modified polyethylene glycol via a thioketal‐based ROS‐cleavable linker, resulting in self‐assembled nanoparticles that remained pharmacologically inactive under systemic conditions. Upon encountering elevated reactive oxygen species within atherosclerotic plaques, the thioketal moiety underwent cleavage, thereby liberating bioactive SV. To enhance plaque‐specific targeting, the delivery system was further functionalized with a fibronectin‐binding peptide, leveraging the pathological overexpression of fibronectin in inflamed endothelial tissues [75].

Increased concentrations of methylglyoxal (MG), a reactive dicarbonyl species, have been associated with the development of atherosclerosis under diabetic conditions [106]. Elevated levels of MG produce ROS, resulting in oxidative stress that contributes to cellular dysfunction and vascular injury [107]. MG has the potential to facilitate atherosclerosis even when hyperglycemia is not present, as evidenced by studies conducted on euglycemic apolipoprotein E knockout mice. In these studies, exposure to MG increased vascular adhesion and atherogenesis, reaching levels comparable to those seen in diabetic states [108]. A study examined the protective role of N‐acetylcysteine (NAC), a cysteine‐based prodrug capable of replenishing intracellular glutathione (GSH), in attenuating MG‐mediated vascular injury. In streptozotocin‐induced diabetic *ApoE^−/−^* mice, NAC treatment markedly decreased aortic plaque burden, MG accumulation, and biomarkers of oxidative stress. At the mechanistic level, NAC promoted MG clearance via GSH‐dependent pathways, upregulated antioxidant enzymes (SOD1, GPx1), and reinstated p‐Akt and p‐eNOS signaling, thereby enhancing endothelial function. In vitro, NAC counteracted MG‐induced oxidative damage in HUVECs, a response negated upon GSH inhibition [109]. These findings underscore the promise of NAC as a redox‐modulating agent for preventing diabetes‐associated atherosclerosis through GSH‐mediated detoxification and vascular protection.

Collectively, systems of ROS‐responsive prodrugs represent a highly effective approach to address the oxidative microenvironment found in atherosclerotic plaques for site‐specific drug activation. These systems consistently improve therapeutic precision by reducing systemic exposure while facilitating controlled intracellular drug release in response to increased ROS levels. However, despite encouraging preclinical results, challenges persist in attaining consistent ROS thresholds across diverse plaque environments, in addition to ensuring long‐term stability and predictable activation kinetics in vivo. These challenges underscore the necessity for more sophisticated ROS‐sensitive chemistries and standardized evaluation models to facilitate clinical translation.

Enzyme‐responsive systems also play a critical role. Smith et al. designed a legumain‐cleavable colchicine prodrug intended to enhance cell‐specific toxicity while minimizing systemic side effects. It has been evaluated only in colorectal cancer and melanoma cell lines; no atherosclerosis model, in vitro or in vivo, has been reported. The prodrug, Suc‐Ala‐Ala‐Asn‐Val‐colchicine, incorporated a tetrapeptide linker cleavable by legumain, a cysteine protease overexpressed in tumors and unstable atherosclerotic plaques. Upon enzymatic cleavage at the Asn residue, the prodrug releases valyl colchicine, a novel cytotoxic agent with improved lipophilicity and reduced off‐target toxicity [110].

Enzyme‐ and receptor‐targeted prodrug platforms offer a biologically sound method for achieving spatially controlled drug activation within atherosclerotic lesions. Nevertheless, the variability in biomarker expression at different stages of plaque development may hinder targeting efficiency, highlighting the necessity for patient‐specific or stage‐adapted therapeutic designs. A better understanding of disease heterogeneity will thus be essential for optimizing these strategies.

pH‐responsive and microenvironment‐sensitive systems further enhance targeting precision by exploiting the acidic conditions characteristic of inflamed tissues. A modular erythrocyte‐derived nanocarrier was developed to enable site‐specific drug delivery to regions of vascular inflammation. This adaptable system incorporates a VCAM‐1‐targeting peptide and a pH‐responsive prodrug into the erythrocyte membrane via DSPE‐PEG‐mediated anchoring, allowing tunable surface functionalization and promoting selective accumulation within atherosclerotic lesions. The acidic milieu characteristic of plaque microenvironments induces prodrug activation, thereby facilitating localized therapeutic release [111].

Overall, these systems illustrate that the integration of biological targeting (for instance, VCAM‐1 recognition) with pH‐responsive activation facilitates more accurate and localized drug delivery. The platform derived from erythrocytes, specifically, gains advantages from biomimicry and modular functionalization, which enhance targeting and minimize off‐target effects. Nevertheless, issues such as fluctuations in tissue pH and the stability of membrane‐integrated components must be resolved to improve clinical applicability.

Early identification and targeted management of atherosclerotic plaques is driven by the interplay between plaque biology, endothelial dysfunction, and impaired nitric oxide (NO) signaling. As plaques enlarge, they progressively restrict blood flow and precipitate ischemic events such as myocardial infarction and stroke. Conventional antiproliferative agents, although effective in suppressing smooth‐muscle proliferation, can delay re‐endothelialization and increase thrombosis risk [112]. Within this pathological environment, NO acts as a central regulator of vascular homeostasis, mediating endothelium‐dependent vasodilation through cGMP signaling and supporting endothelial repair at sites of vascular injury [113]. However, endogenous NO is short‐lived due to rapid scavenging by hemoglobin, limiting its therapeutic utility.

NO‐releasing prodrugs and nanodelivery systems have therefore been developed to restore NO bioactivity and overcome the instability of gaseous NO. These platforms enable controlled NO release in response to diverse stimuli including pH changes, enzymatic activity, ultrasound, photothermal effects, and light irradiation providing spatiotemporal precision unattainable with free NO gas [114–117].

The early identification and management of atherosclerotic plaques is crucial for a variety of reasons. As atherosclerotic plaques continue to accumulate, they may obstruct blood circulation, consequently leading to ischemic vascular conditions such as heart attacks or strokes [112]. Antiproliferative medications have demonstrated a detrimental effect on re‐endothelialization, resulting in an increased risk of thrombosis [118]. Nanomaterials exhibiting catalytic, optical, and thermal functionalities have facilitated the development of diverse therapeutic modalities for atherosclerotic plaque management, including antioxidant approaches, photodynamic therapy (PDT), and photothermal therapy (PTT) [119, 120]. NO, an endogenous gaseous signaling molecule, was initially identified for its regulatory role in a wide range of physiological and pathological functions [121]. NO, functioning as a key intercellular messenger, modulates vascular homeostasis by promoting endothelium‐dependent vasodilation via cyclic guanosine monophosphate (cGMP) signaling pathways [115]. Within this framework, NO serves both as a bioactive mediator and a therapeutic effector, contributing to endothelial regeneration at atherosclerotic lesion sites [19]. A major limitation of this approach lies in the inherent instability and rapid degradation of NO, primarily due to its swift interaction with hemoglobin. To overcome this limitation and enhance therapeutic efficacy, NO prodrugs have been developed to compensate for the insufficient endogenous production of NO by the endothelium. In particular, NO‐releasing nanoprodrugs have emerged as effective substitutes for gaseous NO in biomedical applications, enabling controlled NO delivery in response to a variety of external stimuli, including pH fluctuations, enzymatic activity, ultrasound, photothermal effects, and light irradiation [116, 117, 122–124]. Moreover, the nontargeted biodistribution of NO prodrugs during systemic circulation often results in their widespread dispersion, thereby reducing their effective concentration at pathological sites. Platelets possess membrane‐bound glycoprotein receptors and adhesion molecules that enable selective interaction with macrophage‐derived foam cells and early‐stage atherosclerotic plaques. Owing to this inherent targeting capability, platelet‐based delivery platforms offer a promising strategy for the precise localization of therapeutic agents to atherosclerotic lesions [125, 126]. On the other hand, noninvasive optical imaging techniques have been widely employed for visualizing atherosclerosis, including near‐infrared (NIR) fluorescence imaging, dual/triple photon imaging, photoacoustic imaging (PAI), and upconversion luminescence imaging [127–130]. In recent years, second near‐infrared (NIR‐II, 1000–1700 nm) imaging has emerged as a promising technique for achieving high‐resolution fluorescence visualization of deep biological tissues. This advancement is primarily attributed to its extended emission wavelength, diminished tissue absorption and scattering, and inherently low autofluorescence [131, 132].

A novel platform for activating prodrugs has been developed by immobilizing inducible nitric oxide synthase (iNOS) onto stainless steel (SST) stents. This enzymatically active surface converts the systemically administered prodrug L‐arginine into NO, a powerful anti‐inflammatory and antithrombotic agent. The immobilized iNOS maintained stable catalytic activity for over 6 months and selectively influenced cellular responses. It enhanced the adhesion and proliferation of ECs while suppressing the growth of smooth muscle cells (SMCs) and platelet adhesion [133]. This method illustrates a spatially restricted prodrug activation strategy, wherein the therapeutic transformation takes place directly at the interface of the implant. It could circumvent the limitations associated with systemic NO delivery and facilitate targeted modulation of vascular healing mechanisms. This concept shows potential for the prevention of in‐stent restenosis and thrombosis by means of localized, sustained NO production from an inert precursor.

### 3.3. Nano‐Prodrug Strategies

Emerging evidence underscores notable progress in the development of prodrug and nano‐prodrug strategies aimed at treating atherosclerosis. Nano‐prodrugs refer to pharmacologically inert conjugates that undergo biotransformation into active pharmaceutical ingredients (APIs) within biological environments. These systems exhibit key advantages, including reduced systemic toxicity, enhanced accumulation at pathological sites, and improved pharmacokinetic behavior [134, 135]. Zwitterionic polymers have emerged as a focal point of research in recent years, serving as nano‐prodrug carrier materials. This is attributed to their remarkable hydrophilicity, resistance to protein adsorption, and prolonged circulation within biological systems [136–138]. The zwitterionic polymer OPDEA, characterized by the presence of a tertiary amine nitrogen‐oxygen moiety (N^+^‐O^−^), has demonstrated potential as a nanocarrier system for drug delivery applications based on its zwitterionic properties [139]. Zwitterionic polymers incorporating the N^+^‐O^−^ functional group exhibit promising capabilities for engineering nanocarrier systems, addressing the limitations of conventional zwitterionic nano‐prodrugs, particularly their inability to initiate cellular transcytosis and offering an innovative therapeutic approach for atherosclerosis and related pathologies [140].

Biomimetic nanocarriers improve targeting efficiency by incorporating biological membranes. To address the limitations of traditional drug delivery approaches in atherosclerosis treatment, a biomimetic nanoplatform (RBC/LFP@PMMP) was designed. This construct incorporates a lipid‐targeting fluorophore (LFP) and a ROS‐responsive prednisolone prodrug copolymer (PMPC‐P[MEMA‐co‐PDMA]), which self‐assembles into micellar nanostructures and is subsequently cloaked with red blood cell membranes. The resulting nanoparticles demonstrated extended systemic circulation, minimized premature drug release, and enhanced localization at atherosclerotic plaque sites. Upon exposure to ROS‐enriched inflammatory environments, the system initiated the release of prednisolone and LFP, thereby enabling precise anti‐inflammatory therapy and lipid‐selective fluorescence imaging [51]. A multifunctional nanosystem, RBCM/RAP@ROSELLA, was designed that integrates stimulus‐triggered drug release with biomimetic stealth characteristics. The platform incorporated a ROS‐responsive prodrug of 5‐aminolevulinic acid (ALA) and rapamycin (RAP), enveloped by nanoscale red blood cell membranes to enhance biocompatibility and reduce immune detection. In the presence of elevated hydrogen peroxide concentrations within atherosclerotic lesions, the nanostructure enables site‐specific intracellular drug release. Cell‐based experiments revealed strong suppression of macrophage and vascular SMC proliferation, whereas in vivo evaluation in zebrafish embryos showed no developmental toxicity; no mammalian atherosclerosis model has been reported for this system [141]. Liver X receptors (LXRs) are ligand‐activated transcriptional regulators that play a pivotal role in modulating lipid metabolism and inflammatory signaling, thereby positioning them as promising candidates for atherosclerosis therapy. However, conventional delivery of LXR agonists is frequently associated with nonspecific activity and excessive hepatic lipid deposition. To address these challenges, a study introduced a polydiacetylene‐derived micellar system engineered for lesion‐directed administration of a synthetic LXR agonist prodrug into atherosclerotic plaques. The polydiacetylene micelles exhibited structural uniformity and colloidal stability, enabling passive targeting to atherosclerotic lesions through enhanced permeability and retention (EPR)‐mediated extravasation. Under systemic conditions, the prodrug remains pharmacologically latent and undergoes activation within the inflammatory plaque microenvironment, thereby promoting site‐specific induction of LXR‐responsive transcriptional pathways. In vivo evaluation in ApoE−/− mice revealed optimal circulation dynamics, targeted deposition in vascular lesions, and pronounced immunoregulatory effects, all achieved without perturbing systemic lipid homeostasis [142]. A micellar codelivery system responsive to ROS was developed to synergistically deliver two therapeutic agents, including salvianolic acid A (SAA) and tanshinone IIA (TSIIA). The nanocarrier system was synthesized using amphiphilic polymeric blocks (PEG‐SAA/PLA‐APBA), which spontaneously organized into colloidally stable micelles. These micelles retained pharmacological latency under physiological conditions and underwent oxidative stress‐induced disassembly within the inflammatory microenvironment of atherosclerotic plaques, thereby facilitating the concurrent release of both therapeutic agents. SAA exerted potent antioxidative and anti‐inflammatory activity, whereas TSIIA regulated lipid metabolism and suppressed foam cell formation [143].

Emerging evidence highlights the functional role of integrin *α*9*β*1 as a receptor for osteopontin (OPN), implicating its downstream signaling cascades in the pathogenesis of multiple autoimmune diseases [144, 145]. OPN functions as a key mediator of cellular adhesion and chemotaxis, with markedly elevated expression levels observed in foam cells localized within atherosclerotic plaques [146, 147]. OPN serves as a key regulator in the recruitment and sustained localization of monocyte‐derived macrophages within sites of chronic inflammation, contributing to the advancement of atherosclerosis and the development of unstable plaques [148, 149]. In the context of biomimetic nanotherapeutic platforms targeting atherosclerosis, one strategic approach employs nanocarriers functionalized with cell membranes exhibiting overexpression of *α*9*β*1 integrins (OEM). A self‐assembly strategy that is spontaneous and oriented right‐side‐out for ROS‐responsive nanotherapeutics was developed, leveraging the inherent affinity between phosphatidylserine (PS) and peptides that target PS, to guarantee the proper functional orientation of the membrane. To improve foam cell targeting within atherosclerotic lesions, ROS‐sensitive prodrugs were integrated with engineered cell membranes enriched in integrin *α*9*β*1 (OEM), resulting in the formation of OEM‐coated ETBNPs. Experimental findings from both in vitro and in vivo studies demonstrated the efficacy of these systems in achieving site‐specific drug delivery and stimulus‐responsive release, establishing a flexible framework for biomimetic nanotherapeutic interventions in atherosclerosis [150]. Multifunctional nanoparticles were engineered to selectively respond to hydrogen peroxide‐enriched microenvironments characteristic of atherosclerotic lesions. The nanoplatform incorporated a ROS‐sensitive linker that facilitated regulated drug release under oxidative stress. In addition, it codelivers anti‐inflammatory and lipid‐lowering agents to cooperatively attenuate plaque progression. Analytical characterization revealed consistent particle morphology, colloidal stability, and peroxide‐induced disassembly. Cellular assays confirmed efficient uptake, reduced oxidative injury, and significant downregulation of inflammatory cytokine expression [151]. An innovative prodrug‐based nanocarrier system was developed to leverage the intrinsic transition of systemic monocytes into lesion‐infiltrating macrophages. This differentiation‐dependent mechanism enabled localized therapeutic activation and facilitated immunological reconditioning within atherosclerotic plaques. The nanoprodrug was engineered to remain inactive under physiological conditions and undergoes activation specifically during the cellular transition from circulating monocytes to macrophages, a process induced by the inflammatory microenvironment of atherosclerotic plaques. This activation pathway was facilitated through legumain, a protease whose expression is elevated during macrophage differentiation. Upon proteolytic cleavage, [152], a pharmacologically active molecule recognized for its potent anti‐inflammatory and immunomodulatory effects in various diseases including atherosclerosis [153–156].

Recent progress in NIR‐II (1000–1700 nm) fluorescence imaging has led to the development of a set of photoresponsive NO prodrugs (RBT‐NO), supported by NIR‐II dyes (RBT‐NH), which are specifically responsive to 808 nm laser irradiation within the clinically relevant therapeutic range. RBT3‐NO exhibited enhanced NO release capacity and notable fluorescence signal amplification. Leveraging this outcome, a biomimetic nano‐prodrug construct (RBT3‐NO‐PEG@PM) was formulated by integrating DSPE‐mPEG5k with platelet membrane components, enabling site‐specific imaging and treatment of atherosclerotic plaques in mouse models [157].

A minimalist nanocomplex (MNC) was developed to concurrently restore endothelial barrier function while inhibiting inflammatory signaling, aimed at targeted vascular therapy. The nanocomplex was formulated from a positively charged polymer matrix and inflammation‐modulating compounds, yielding a condensed nanoscale assembly that preferentially localizes to inflamed vascular regions. It reinforced endothelial barrier function by promoting the expression of tight junction components and concurrently suppresses NF‐*κ*B‐dependent proinflammatory signaling pathways. Experimental assays demonstrated reduced endothelial permeability and attenuated cytokine release, whereas in vivo evaluation in murine models confirmed selective vascular accumulation, decreased extravasation, and substantial mitigation of inflammation‐induced vascular injury [158].

Biomimetic nano‐prodrug platforms signify promising progress in the treatment of atherosclerosis by enhancing circulation stability, facilitating immune evasion, and enabling lesion‐specific targeting via the incorporation of natural cell membranes. These approaches enhance drug accumulation at affected sites while minimizing clearance by the reticuloendothelial system. However, challenges such as variability in membrane sourcing, possible immunogenicity, and the scalability of manufacturing processes continue to pose significant obstacles to clinical application. Future advancements in membrane engineering and large‐scale production techniques will be crucial for the complete realization of their therapeutic capabilities.

## 4. Multifunctional and Theranostic Platforms

Recent progress has resulted in the creation of multifunctional systems that unify therapy, targeting, and imaging into a single platform [159–161]. Theranostic nanoplatforms merge diagnostic and therapeutic capabilities and have been successfully utilized in various diseases and pathological states [162–164].

Sterol‐conjugated succinobucol derivatives constitute a promising category of multifunctional prodrugs, offering potential for a safer and more effective approach to the treatment of atherosclerosis. A collection of succinobucol‐sterol conjugates was formed by covalently attaching succinobucol to sterols and stanols derived from plants, including stigmastanol, *β*‐sitosterol, stigmasterol, and cholesterol. The rationale behind this conjugation approach was to improve gastrointestinal uptake through sterol‐mediated lipophilicity, facilitate targeted delivery to lipid metabolic pathways via enterohepatic circulation, and integrate the hypocholesterolemic properties of sterols with the antioxidant efficacy of succinobucol. The resulting conjugates demonstrated advantageous physicochemical characteristics, including acid‐resistant structural integrity and well‐defined crystalline architecture. Cell viability assessments indicated lower cytotoxicity relative to succinobucol alone, whereas DPPH‐based antioxidant profiling yielded antiradical power (ARP) values on par with or surpassing those of ascorbic acid [165].

Zhang et al. created a versatile nanoparticle system utilizing copper oleate nanoparticles (CuNPs) for combined PAI and targeted antiangiogenic treatment. In this platform, CuNPs were stabilized with phospholipid surfactants and manipulated with an *α*v*β*3‐integrin‐targeting ligand to facilitate selective accumulation in angiogenic vascular areas. A lipase‐sensitive fumagillin prodrug (Fum‐PD), strategically placed at the Sn2 position of phospholipids, was integrated into the nanoparticle lipid layer, permitting enzymatic activation in pathological microenvironments. This innovative design allowed for concurrent improvement of diagnostic imaging and localized therapeutic release. In a Matrigel‐based murine angiogenesis model, *α*v*β*3‐targeted CuNPs exhibited significant photoacoustic signal amplification and markedly decreased neovascular formation following the enzymatic activation of the Fum‐PD [166].

Multifunctional and theranostic nanoplatforms merge therapeutic delivery with diagnostic imaging, facilitating concurrent treatment and real‐time observation of atherosclerotic development. These platforms show considerable promise for precision medicine through integrating stimuli‐responsive drug release with imaging techniques such as PAI, NIR fluorescence, and NIR‐II visualization. Together, they provide enhanced spatial‐temporal regulation of therapy and make important insights into treatment effectiveness. However, the heightened structural complexity of these systems presents notable obstacles regarding regulatory approval, manufacturing scalability, and long‐term biosafety evaluation. Furthermore, achieving a balance between multifunctionality and clinical practicality is an important challenge, as excessively intricate systems may impede translational viability despite their robust preclinical outcomes.

**Figure 4:**
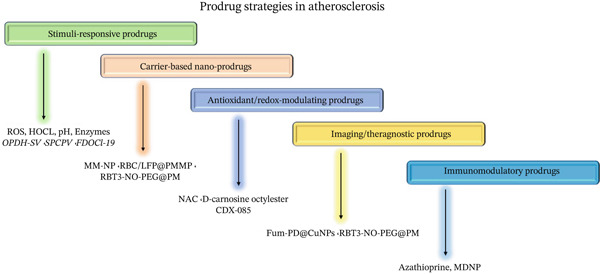
Categorization of prodrug strategies in atherosclerosis therapy. The diagram illustrates six major classes of prodrug platforms, including stimuli‐responsive systems (e.g., ROS‐, HOCl‐, pH‐, and enzyme‐activated), carrier‐based nano‐prodrugs (e.g., macrophage, RBC, and platelet membrane‐coated), antioxidant/redox‐modulating agents, imaging‐enabled theranostic constructs, and immunomodulatory prodrugs. Representative examples such as OPDH‐5V, FDOC‐19, and MM‐NP are highlighted to demonstrate the diversity of activation mechanisms and delivery modalities.

Figure 4 presents a systematic categorization of prodrug‐based therapeutic strategies under development for the treatment of atherosclerosis. These strategies encompass stimuli‐responsive constructs reactive to ROS, HOCl, pH fluctuations, or enzymatic activity as well as carrier‐mediated nanoformulations, redox‐modulating agents, theragnostic platforms, and immunoregulatory compounds. Representative examples within each class demonstrate the breadth and functional specificity of contemporary prodrug engineering aimed at mitigating the progression of atherosclerotic disease.

Table 1 summarizes recent advances in prodrug platforms designed for targeted atherosclerosis therapy.

**Table 1:** Overview of prodrugs for targeted atherosclerosis therapy: Composition, in vitro performance, and in vivo efficacy.

Prodrug	Chemical composition/drug	Preclinical in vitro findings	Animal model/experimental model	Preclinical in vivo findings	Ref
AEE	Esterified conjugate of aspirin and eugenol; phenolic hydroxyl and carboxyl groups masked to enhance stability and reduce GI toxicity	—	Syrian golden hamsters (HFD‐induced atherosclerosis model)	Reversed HFD‐induced biochemical disturbances (↓TG, ↓TC, ↓LDL, ↓AST, ↓LDH, ↓AI), improved hepatic histology, reduced body‐weight gain and atherosclerosis index, enhanced aortic structure and lumen area, and normalized lipid, amino acid, and energy metabolism pathways.	[81]
ETC‐1002	Liver‐activated prodrug converted to ETC‐1002‐CoA; inhibits ACLY; can activate AMPK*β*1, but antiatherosclerotic effects are AMPK‐independent	ETC‐1002‐CoA competitively inhibits ACLY; selective hepatic activation due to ACSVL1; no activation in skeletal muscle, no myotoxicity.	ApoE−/− and ApoE−/−/AMPK*β*1−/− (DKO) mice	Reduced hepatic cholesterol and triglycerides; suppressed lipogenesis; ~40% LDL‐C reduction via SREBP2‐mediated LDLR upregulation; attenuated atherosclerosis independent of AMPK; no myotoxicity.	[85]
ETC‐1002	Liver‐specific prodrug converted to ETC‐1002‐CoA via ACSVL1; inhibits ACLY	In primary hepatocytes, ETC‐1002‐CoA inhibited fatty acid and sterol synthesis, enhanced fatty acid oxidation, and reduced inflammatory cytokine production (IL‐6, IL‐8, CCL2, CXCL10). No cytotoxicity observed in skeletal muscle cells.	Ldlr−/− mice, ApoE−/− mice, Yucatan miniature pigs	Reduced LDL‐C, triglycerides and aortic lesion area; promoted M2 macrophage polarization; reduced M1 macrophage markers; improved glucose tolerance; antiatherosclerotic efficacy remained independent of AMPK signaling.	[86]
Azathioprine (converted to 6‐MP)	Azathioprine as a prodrug rapidly metabolized to 6‐mercaptopurine (6‐MP), a purine synthesis inhibitor	Induces apoptosis in THP‐1 monocytes via downregulation of Bcl‐2 and Bcl‐XL; inhibits adhesion by suppressing PECAM‐1 and VLA‐4; reduces MCP‐1 expression and secretion in activated macrophages.	ApoE∗3‐Leiden transgenic mice	Local delivery via drug‐eluting cuff significantly reduced atherosclerotic lesion size (up to 85.9%), decreased MCP‐1 levels, increased vascular apoptosis, and reduced macrophage infiltration in plaques. No systemic toxicity observed with dietary administration.	[90]
A‐002 (Varespladib methyl)	Orally bioavailable prodrug of A‐001, a potent inhibitor of sPLA2	Inhibits sPLA2 groups IIA, V, and X with low nanomolar IC50 values; suppresses PLA2 activity in transgenic mice overexpressing human sPLA2 IIA.	ApoE−/− mice (standard and angiotensin II‐accelerated models)	Reduced aortic atherosclerosis by 40%–50%; lowered total cholesterol; prevented AngII‐induced aneurysm formation; sustained inhibition of sPLA2 activity In VISTA‐16, varespladib increased myocardial infarction (3.4% vs. 2.2%; HR 1.66) and the trial was stopped early; sPLA2 inhibition was subsequently abandoned.	[94]
CDX‐085	Esterified water‐soluble prodrug of astaxanthin	—	Beagle dogs (pharmacokinetic study); LDLR−/− and ApoE−/− mice	Pharmacokinetic studies demonstrated ~10‐fold higher plasma astaxanthin compared with the parent compound. In LDLR−/− and ApoE−/− mice, CDX‐085 reduced total cholesterol, triglycerides, and atherosclerotic lesions while improving antioxidant distribution among lipoproteins.	[95]
D‐carnosine octylester	Esterified form of D‐carnosine designed for oral bioavailability; hydrolyzed by esterases to release active D‐carnosine	Attenuated HNE‐induced cytotoxicity, apoptosis, and ROS generation in VSMCs; no effect on H_2_O_2_‐induced stress.	ApoE−/− mice fed a Western diet	Reduced aortic lesion area (−18%), necrotic core (−54%), and inflammation; increased plaque stability and collagen (+48%); attenuated renal fibrosis, apoptosis, and oxidative stress; elevated urinary CAR‐HNE levels confirmed prodrug activation.	[97]
MM‐NP	ROS‐sensitive linker conjugated to anti‐inflammatory and lipid‐modulating agents; core cloaked with macrophage‐derived membrane	ROS‐triggered drug release; significant suppression of TNF‐*α* and IL‐6; enhanced cholesterol efflux via ABCA1/ABCG1 upregulation; low cytotoxicity and high cellular uptake.	ApoE−/− mice	Targeted accumulation in atherosclerotic plaques; reduced plaque area and lipid deposition; prolonged circulation; minimal systemic toxicity; dual modulation of inflammation and lipid metabolism.	[104]
OPDH‐SV	Covalent linkage of simvastatin to a zwitterionic polymer via oxalate bonds	Facile intercellular movement via the endocytosis‐efflux pathway.	ApoE−/− mice	OPDH‐SV demonstrated strong therapeutic efficacy and safety while reducing drug cytotoxicity and maintaining long‐term circulation. It effectively enriched atherosclerotic plaques through an “endocytosis‐efflux” mechanism.	[105]
PCPV (ROS‐responsive VCAM‐1‐targeted prodrug micelle)	Simvastatin covalently linked to polymer via peroxalate ester bonds; micelle functionalized with VCAM‐1‐targeting ligand	ROS‐triggered release of active SV; efficient cellular uptake; suppression of inflammatory cytokines; enhanced cholesterol efflux.	ApoE−/− mice	Prolonged circulation; selective accumulation in atherosclerotic plaques; reduced lipid deposition and vascular inflammation; minimal systemic toxicity.	[74]
TPTS‐FBP (fibronectin‐targeted ROS‐responsive simvastatin prodrug)	Simvastatin conjugated to *α*‐tocopherol–PEG via thioketal linker; functionalized with FBP	ROS‐triggered drug release; efficient cellular uptake; suppression of inflammatory cytokines; minimal cytotoxicity.	ApoE−/− mice	Targeted accumulation in atherosclerotic plaques; reduced lipid deposition and inflammation; prolonged circulation; enhanced therapeutic efficacy.	[75]
NAC	Cysteine‐based antioxidant prodrug that replenishes intracellular GSH	In MG‐treated HUVECs, NAC reduced ROS levels, restored antioxidant enzymes (SOD‐1, GPX‐1), and reactivated p‐Akt/p‐eNOS signaling. Protective effects were abolished by GSH inhibition (BSO).	Streptozotocin‐induced diabetic ApoE−/− mice fed a high‐lipid diet	NAC significantly reduced atherosclerotic plaque size, corrected MG and protein carbonyl accumulation, decreased serum MDA, increased aortic GSH, SOD‐1, GPX‐1, and restored p‐Akt/p‐eNOS signaling and serum NO levels.	[109]
Legumain‐cleavable colchicine prodrug	Suc‐Ala‐Ala‐Asn‐Val‐colchicine (colchicine conjugated to a legumain‐cleavable tetrapeptide)	Selective cytotoxicity in legumain‐overexpressing cancer cell lines (HCT116, M38L); reduced toxicity in low‐legumain lines (SW620, M4C); cleavage confirmed via HPLC; inhibition by cystatin E/M and Dyngo‐4a indicates dependence on legumain activity and endocytosis.	—	—	[110]
Erythrocyte‐FBP (plug‐and‐play erythrocyte nanoplatform)	Erythrocyte membrane functionalized with VCAM‐1‐targeting peptide and acid‐sensitive prodrug via DSPE‐PEG conjugation	Selective binding to activated endothelial cells through VCAM‐1 targeting, acid‐responsive drug release, enhanced cellular uptake, and low cytotoxicity.	ApoE−/− mice fed a high‐fat diet	Prolonged blood circulation, enhanced accumulation in atherosclerotic plaques, targeted acid‐responsive drug release, significant reduction in plaque burden, lipid deposition, and inflammatory responses, with good biocompatibility and no obvious systemic toxicity.	[111]
iNOS‐immobilized SST surface	L‐arginine (prodrug) converted to NO via immobilized iNOS on stainless steel stents	Enhanced EC adhesion and proliferation; inhibited SMC growth and platelet adhesion; stable NO production for 6 months.	—	—	[133]
OEM‐coated ETBNPs	Self‐assembly using PS‐peptide affinity to ensure correct membrane orientation	Right‐side‐out membrane orientation; sustained drug‐release behavior; inhibited foam‐cell lipid uptake, promoted intracellular lipid efflux, and regulated macrophage phenotypic conversion.	ApoE−/− mice	Enhanced targeted accumulation in atherosclerotic plaques with on‐demand drug release, resulting in improved therapeutic efficacy against pathological vascular remodeling.	[150]
H_2_O_2_‐NP (dual‐function hydrogen peroxide‐responsive nanoparticles)	Nanoparticles with H_2_O_2_‐cleavable linker; coloaded with anti‐inflammatory and lipid‐lowering agents	H_2_O_2_‐responsive drug release, efficient macrophage targeting, fluorescence imaging capability, prevention of burst drug release, and improved therapeutic efficacy of the loaded drug.	ApoE−/− mice	Targeted accumulation and imaging of atherosclerotic plaques, enhanced antiatherosclerotic efficacy, and good biocompatibility with minimal systemic toxicity.	[151]
RBT3‐NO‐PEG@PM	RBT3‐NO prodrug encapsulated in DSPE‐mPEG5k micelles and coated with platelet membrane (PM)	High NO‐release efficiency (81.2%), NIR‐II fluorescence activation (933 nm), excellent photostability, low cytotoxicity (> 80% cell viability), and efficient intracellular NO release in RAW264.7 macrophages and HUVECs.	ApoE−/− mice	Targeted accumulation at atherosclerotic plaques, reduced lipid deposition, attenuated inflammatory cytokines (TNF‐*α*, MCP‐1, IL‐8, and TGF‐*β*), promoted endothelial cell migration, delayed plaque progression, and demonstrated good biocompatibility.	[157]
MNC	Polysialic acid‐based bi‐prodrug nanocomplex coloaded with budesonide and L‐arginine	Selectively targeted activated endothelial cells via E‐selectin; enhanced eNOS expression and NO production; reinforced endothelial junctions; stabilized I*κ*B*α*; suppressed NF‐*κ*B signaling; reduced inflammatory cytokine secretion; improved endothelial barrier integrity.	ApoE−/− mice	Selectively accumulated in inflamed vasculature, reduced atherosclerotic plaque burden and vascular inflammation, decreased inflammatory cell infiltration, improved vasodilation, and demonstrated good biocompatibility.	[158]
Succinobucol–sterol conjugates	Covalent esterification of succinobucol with plant sterols (stigmastanol, *β*‐sitosterol, stigmasterol) and cholesterol	Low cytotoxicity towards mouse fibroblasts and potent antioxidant activity (ARP comparable to or greater than ascorbic acid)	—	—	[165]
Fum‐PD@CuNPs	Sn2 lipase‐labile fumagillin prodrug esterified with 1‐palmitoyl‐2‐azelaoyl‐sn‐glycero‐3‐phosphocholine and incorporated into *α*v*β*3‐targeted copper nanoparticles	Stable nanoparticle formulation with preserved particle size and zeta potential, high photoacoustic contrast, and Sn2 phospholipase‐responsive prodrug activation.	Nude mice (Matrigel plug angiogenesis model; nonatherosclerosis model)	Targeted inhibition of neovessel sprouting, reduced photoacoustic signal following treatment, and proof‐of‐concept for *α*v*β*3‐targeted photoacoustic‐guided lipase‐responsive drug delivery.	[166]

Abbreviations: ACL, ATP‐citrate lyase; AEE, aspirin eugenol ester; ALA, aminolevulinic acid; ApoE, atherosclerosis‐prone apolipoprotein E‐deficient; EC, endothelial cell; EPR, enhanced permeability and retention; ETC‐1002, bempedoic acid; FBP, fibronectin‐binding peptide; Fum‐PD, fumagillin prodrug; GSH, glutathione; iNOS, inducible nitric oxide synthase; MDNP, monocyte differentiation‐activated nanoprodrug; MM‐NP, macrophage membrane‐coated nanoprodrug; MNC, minimalist nanocomplex; NAC, N‐acetylcysteine; NO, nitric oxide; RAP, rapamycin; ROS, reactive oxygen species; SAA, salvianolic acid A; SMC, smooth muscle cell; sPLA2, secretory phospholipase A2; TSIIA, tanshinone IIA; VSMCs, vascular smooth muscle cells.

## 5. Concluding Remarks and Future Perspectives

Atherosclerosis represents a biologically intricate vascular condition driven by persistent oxidative imbalance, inflammation, and disruptions in lipid metabolism [167]. Although conventional interventions such as statins, angiotensin‐converting enzyme inhibitors, and revascularization procedures have been extensively utilized, their therapeutic efficacy is frequently constrained by inadequate plaque targeting, systemic adverse effects, and inefficient pharmacokinetic behavior [168]. In response to these limitations, the development of prodrug and nano‐prodrug platforms introduces a novel therapeutic framework that enhances site‐specific drug delivery, facilitates stimulus‐responsive activation, and improves overall safety profiles in the management of atherosclerotic disease [169].

Prodrugs are structurally engineered to retain therapeutic latency until they are exposed to defined pathological cues, including elevated levels of ROS, acidic microenvironments, or enzymatic activity within atherosclerotic lesions [170]. Stimulus‐responsive activation mechanism significantly reduces unintended cytotoxicity and improves site‐specific pharmacological accuracy [171]. Expanding on this methodology, nano‐prodrug systems utilize sophisticated delivery platforms, including surface modifications, zwitterionic polymers, and membrane coatings derived from macrophages or erythrocytes aimed at integrin targeting [172–175]. These design approaches are intended to extend systemic circulation time, evade immune detection, and improve selective accumulation at pathological vascular sites.

Compared with traditional pharmacological agents, nano‐prodrug formulations demonstrate enhanced therapeutic performance through bimodal pathways, notably the regulation of lipid metabolism, attenuation of inflammatory responses, and inhibition of foam cell formation. Their structural integrity is maintained through the incorporation of covalent linkers, camouflaging surface modifications, and regulated release systems that collectively mitigate early breakdown and ensure sustained bioavailability. Furthermore, biocompatibility is significantly enhanced, as evidenced by reduced systemic drug exposure and minimized immunogenic reactions in various in vivo experimental models.

Integrated nanoplatforms such as RBCM/RAP@ROSELLA, OPDH‐SV, and SPCPV highlight the potential for unifying diagnostic imaging, stimuli‐responsive drug liberation, and biologically inspired targeting strategies within a single therapeutic construct. These advanced systems effectively attenuate plaque progression while concurrently enabling dynamic assessment of pharmacological performance. Furthermore, ligand‐mediated targeting mechanisms utilizing moieties such as VCAM‐1, *α*v*β*3‐integrin, and *α*9*β*1‐integrin combined with ROS‐sensitive linkers have demonstrated efficacy in facilitating localized drug activation and minimizing unintended tissue exposure.

Despite recent progress, numerous obstacles persist in the clinical translation of nano‐prodrug technologies. Key barriers include manufacturing scalability, experimental consistency, sustained biological compatibility, and adherence to regulatory standards [176, 177]. Moreover, variability in plaque‐associated tissue landscapes and individual differences in oxidative stress profiles among patients underscore the need for tailored formulation approaches. Effective clinical implementation of these nanotherapeutics further demands comprehensive pharmacodynamic simulations, biomarker‐informed stratification strategies, and economically viable production processes to ensure therapeutic accessibility and reproducibility [30, 178].

From a forward‐looking perspective, the advancement of prodrug‐based nanotherapeutics for atherosclerosis is contingent upon several key developmental pathways that may facilitate clinical implementation and optimize therapeutic outcomes. No nano‐prodrug platform for atherosclerosis has yet entered a clinical trial; the evidence reviewed above is entirely preclinical and predominantly derived from ApoE−/− mice. Well‐structured Phase I and II evaluations are therefore imperative to establish long‐term safety, pharmacokinetic reliability, and therapeutic efficacy in humans, especially given the complex behavior of nanoscale constructs in biological systems. Customized therapeutic delivery modalities guided by patient‐specific biomarkers and supported by advanced imaging technologies offer promising avenues for addressing heterogeneity in plaque morphology and oxidative stress profiles. The adoption of multifunctional systems that integrate therapeutic action, diagnostic imaging, and real‐time performance monitoring holds significant potential to transform atherosclerosis management. Furthermore, the design of multistimuli‐responsive biomaterials capable of reacting to oxidative stress, pH fluctuations, and enzymatic activity may substantially improve targeting specificity while minimizing unintended interactions. Establishing comprehensive regulatory infrastructures tailored to the unique pharmacological and structural attributes of nano‐prodrugs will be essential to ensure safety, reproducibility, and scalability in clinical contexts. Collectively, these future directions highlight the capacity for paradigm‐shifting innovation in cardiovascular precision therapy.

**Nomenclature**
ABCA1: ATP‐binding cassette transporter A1
ABCG1: ATP‐binding cassette transporter G1
ALA: 5‐aminolevulinic acid
API: active pharmaceutical ingredient
ApoE: apolipoprotein E
ARP: antiradical power
CD36: cluster of differentiation 36
cGMP: cyclic guanosine monophosphate
DPPH: 2,2‐diphenyl‐1‐picrylhydrazyl
EC: endothelial cell
EPR: enhanced permeability and retention
GSH: glutathione
HDL: high‐density lipoprotein
HL‐60: human promyelocytic leukemia cell line
HMG‐CoA: 3‐hydroxy‐3‐methylglutaryl coenzyme A
HOCl: hypochlorous acid
HUVEC: human umbilical vein endothelial cell
iNOS: inducible nitric oxide synthase
LD‐CP: low‐density cholesterol‐like particle
LDL: low‐density lipoprotein
LFP: lipid‐targeting fluorophore
LXR: liver X receptor
MEMA: 2‐methoxyethyl methacrylate
MG: methylglyoxal
MMP: matrix metalloproteinase
MPO: myeloperoxidase
NAC: N‐acetylcysteine
NF‐*κ*B: nuclear factor kappa B
NIR: near‐infrared
NIR‐II: second near‐infrared window
NO: nitric oxide
OEM: osteopontin receptor‐overexpressing engineered membrane
OPN: osteopontin
PAI: photoacoustic imaging
PD: prodrug
PDT: photodynamic therapy
PEG: polyethylene glycol
PLA: polylactic acid
PLGA: poly(lactic‐co‐glycolic acid)
PMPC: poly(2‐methacryloyloxyethyl phosphorylcholine)
PS: phosphatidylserine
PTT: photothermal therapy
RAP: rapamycin
RBC: red blood cell
RBCM: red blood cell membrane
RCS: reactive carbonyl species
ROS: reactive oxygen species
SAA: salvianolic acid A
SMC: smooth muscle cell
SOD1: superoxide dismutase 1
SPCPV: ROS‐responsive simvastatin prodrug micelle
SST: stainless steel
SV: simvastatin
TG: triglyceride
TSIIA: tanshinone IIA
VCAM‐1: vascular cell adhesion molecule‐1
VLDL: very‐low‐density lipoprotein.

## Author Contributions

Conceptualization: E.M. and A.S.; investigation: E.M., W.A., P.K., and A.S.; writing—original draft: E.M.; writing—review and editing: W.A., A.S., and P.K.; supervision: P.K. and A.S.

## Funding

No funding was received for this manuscript.

## Ethics Statement

The authors have nothing to report.

## Consent

The authors have nothing to report.

## Conflicts of Interest

The authors declare no conflicts of interest.

## Data Availability

Data sharing is not applicable to this article as no datasets were generated or analyzed during the current study.
